# Intramolecular Charge Transfer of Curcumin and Solvation Dynamics of DMSO Probed by Time-Resolved Raman Spectroscopy

**DOI:** 10.3390/ijms23031727

**Published:** 2022-02-02

**Authors:** Myungsam Jen, Sebok Lee, Gisang Lee, Daedu Lee, Yoonsoo Pang

**Affiliations:** Department of Chemistry, Gwangju Institute of Science and Technology, 123 Cheomdangwagi-ro, Buk-gu, Gwangju 61005, Korea; watqdt@gist.ac.kr (M.J.); leesebok@gist.ac.kr (S.L.); rltkd0603@gist.ac.kr (G.L.); leedd1896@gist.ac.kr (D.L.)

**Keywords:** intramolecular charge transfer, excited-state dynamics, solvation dynamics, femtosecond stimulated Raman spectroscopy, hydrogen bonding

## Abstract

Intramolecular charge transfer (ICT) of curcumin in dimethyl sulfoxide (DMSO) solution in the excited state was investigated by femtosecond electronic and vibrational spectroscopy. Excited-state Raman spectra of curcumin in the locally-excited and charge-transferred (CT) state of the S_1_ excited state were separated due to high temporal (<50 fs) and spectral (<10 cm^−1^) resolutions of femtosecond stimulated Raman spectroscopy. The ultrafast (0.6–0.8 ps) ICT and subsequent vibrational relaxation (6–9 ps) in the CT state were ubiquitously observed in the ground- and excited-state vibrational modes of the solute curcumin and the ν_CSC_ and ν_S=O_ modes of solvent DMSO. The ICT of curcumin in the excited state was preceded by the disruption of the solvation shells, including the breakage of hydrogen bonding between curcumin and DMSO molecules, which occurs at the ultrafast (20–50 fs) time scales.

## 1. Introduction

**Figure 1:**
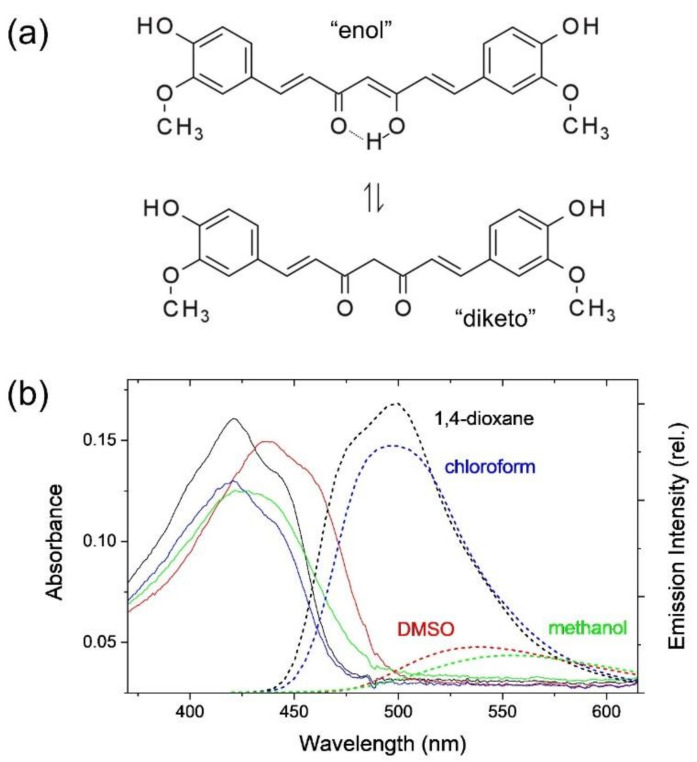
(**a**) Molecular structure of (1*E*,6*E*)-1,7-bis(4-hydroxy-3-methoxyphenyl)hepta-1,6-diene-3,5-dione (curcumin) in enol and diketo forms, (**b**) absorption (solid lines) and emission (dotted lines) spectra of curcumin in 1,4-dioxane, chloroform, DMSO, and methanol solutions. A relative (rel.) intensity scale was used for the emission spectra.

(*E,E*)-1,7-Bis(4-Hydroxy-3-methoxyphenyl)-1,6-heptadiene-3,5-dione (curcumin) is one of the natural pigments often used as an antioxidant, anti-inflammatory, and anti-cancer agent, and has thus attracted interest from researchers in chemistry and related disciplines [1,2,3]. Curcumin is also the primary ingredient of turmeric and curry powders and has been recognized as one of the multi-functional therapeutic compounds for neurodegenerative diseases, cardiovascular, malignancies, etc. [4]. Curcumin may exist either in diketo or enol form as expected from the molecular structures shown in Figure 1a. Many experimental and theoretical studies have confirmed that the enol form exists predominantly in the gas phase and various solution phases in a wide polarity range [5,6,7,8,9,10,11]. The enol form of curcumin may undergo the intramolecular proton transfer reaction in the hydrogen-bonded chelate center, resulting in the identical (symmetric) enol form as the product. The intramolecular proton (hydrogen) transfer reaction in the excited state, which may occur in ultrafast time scales of a few hundred femtoseconds, has been suggested responsible for the efficient non-radiative deactivation of the excited states [6,12,13,14,15]. However, the spectral changes related to the intramolecular proton transfer reaction of curcumin may not be observed due to the symmetric nature of the molecular structure.

Excited-state dynamics of curcumin has been extensively studied by ultrafast transient absorption and fluorescence spectroscopy [5,12,13,16,17,18,19,20,21]. The lifetimes and quantum yields of curcumin strongly increase with the increase of the solvent polarity in general, which is seemingly related to the changes in the intramolecular hydrogen bonding of the enol form of curcumin in solution [17,22]. In some polar solvents with the hydrogen bond donating and accepting groups, much shorter lifetimes and smaller quantum yields were observed from the fluorescence kinetic measurements compared to the cases in the solvents of similar polarity. The increased solvation dynamics via the intermolecular hydrogen bonding facilitates the non-radiative decay of the excited states of curcumin [13,14,15,17]. Additional ultrafast excited-state dynamics of curcumin representing the intramolecular charge transfer (ICT) in the S_1_ state were observed in the transient absorption measurements in methanol, dimethylformamide, and dimethyl sulfoxide (DMSO) [5]. Overall, the excited-state dynamics of curcumin in solution, including the intramolecular proton transfer and charge transfer processes, are strongly perturbed by the solvent dynamics via the week dipole–dipole interactions or the strong hydrogen-bonding interactions. However, the detailed structural changes of curcumin during the ICT process in the S_1_ state or the solvation dynamics via hydrogen bonding facilitating the deactivation of the photo-excited curcumin have not been experimentally implemented, especially by time-resolved vibrational spectroscopy.

Femtosecond stimulated Raman spectroscopy (FSRS) is a powerful spectroscopic tool for the investigation of vibrational structures of analytes with both high temporal (<50 fs) and spectral (<10 cm^−1^) resolutions [23,24]. FSRS has been successfully applied to various chemical and biological systems where the population or frequency changes of multiple vibrational modes in the fingerprint region reveal ultrafast structural dynamics in the excited states or the structural dynamics of fluorescent proteins upon metal-ion binding [25,26,27,28,29,30]. When combined with electronic spectroscopic methods, including transient absorption and fluorescence upconversion, FSRS can be a useful experimental tool for the structural changes of a molecule accompanying ultrafast electronic transitions in the state [31,32]. Furthermore, the vibrational modes of the reactant and solvent molecules can be separately observed in FSRS due to its wide spectral coverage and high spectral resolution. The FSRS has successfully investigated ultrafast excited-state dynamics with the structural changes and solvation dynamics [33,34].

In this work, the excited-state dynamics of curcumin and the solvation dynamics of DMSO have been investigated by femtosecond transient absorption and FSRS. The stimulated Raman probe of high temporal resolution provides key structural information of probe molecules during the ICT process in the S_1_ state and the preceding and subsequent vibrational relaxations. The ultrafast changes in the ν_S=O_ and ν_CSC_ modes of solvent DMSO will also be used to explain the solvation dynamics of DMSO before and during the ICT process of curcumin in the excited states.

## 2. Results and Discussion

### 2.1. Intramolecular Charge Transfer of Curcumin

Figure 1b shows the absorption and emission spectra of curcumin in 1,4-dioxane, chloroform, DMSO, and methanol solutions. The absorption maximum appears at 421 nm with another shoulder bands at ~440 nm in the weakly polar solvents of 1,4-dioxane and chloroform. It appears that the absorption band of curcumin is red-shifted with the increase of solvent polarity; 425 and ~440 nm (shoulder) in methanol and 437 and 458 nm (shoulder) in DMSO. The red-shifts of the absorption band with the increase of the solvent polarity (compared to 408 nm in cyclohexane) and the growth of the shoulder bands in the longer wavelengths may represent further complicated solute-solvent interactions including intra- and inter-molecular hydrogen bonding [5,12]. On the other hand, the emission bands appearing at 498 nm in 1,4-dioxane and chloroform are strongly red-shifted in DMSO (538 nm) and methanol (555 nm) with the apparent decreases in the fluorescence quantum yields. The Stokes shifts of curcumin in nonpolar solvent (1500 cm^−1^ in cyclohexane [12]) increase with the increase of solvent polarity. However, the strong increases in DMSO (4300 cm^−1^) and methanol (5510 cm^−1^) compared to those in 1,4-dioxane and chloroform (3670 cm^−1^) clearly show that the photophysical properties of curcumin in these polar solvents are strongly related to the hydrogen bond capacity rather than the solvent polarity. Methanol is known as both a strong hydrogen bond donor (α = 0.93) and acceptor (β = 0.62) and DMSO as a strong hydrogen bond acceptor (β = 0.76) [17,35]. Compared to methanol and DMSO, the hydrogen bond capacities of 1,4-dioxane (β = 0.37) and chloroform (α = 0.44) appear quite small [36]. The red-shifts in the emission bands and the decreases in the quantum yields have been considered evidence for the ICT in polar solvents with the proposed strong dipole moment increase (Δμ~6.1 D) in the S_1_ excited state [12].

### 2.2. Excited-State Dynamics of Curcumin by Transient Absorption Spectroscopy

**Figure 2:**
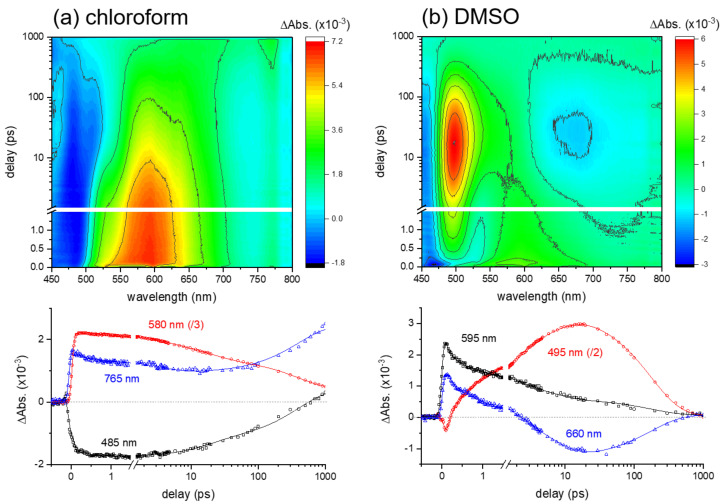
Surface plots and kinetics of excited-state absorption (ESA) and stimulated emission (SE) bands in transient absorption results of curcumin in (**a**) chloroform and (**b**) dimethyl sulfoxide (DMSO) obtained with 403 nm excitation.

Transient absorption results of curcumin in chloroform and DMSO obtained with 403 nm excitation are compared in Figure 2. The differences in the excited-state dynamics in two polar aprotic solvents are displayed in the surface plots. The kinetic traces of the major excited-state absorption and stimulated emission bands are shown, together with the global fit analyses with the convoluted functions of exponential decay and the instrument response function (IRF) Gaussian [33,37].

The excited-state dynamics of curcumin in a weak hydrogen-bond donor, chloroform is relatively simple. Both the excited-state absorption (ESA) band centered at 580 nm and the stimulated emission (SE) band centered at 485 nm show multiexponential rises and decays with the time constants of 0.79 ± 0.04, 6.3 ± 0.2, and 210 ± 7 ps. Similar to the results obtained in a weak hydrogen-bond acceptor, 1,4-dioxane (see Figure S4 in the Supplementary Materials), the fastest component (0.79 ps) represents the vibrational relaxation in the locally-excited (LE) state, which has been reported as being strongly dependent on the solvation dynamics [5,14,16]. Two decay components of 6.3 and 210 ps in chloroform solution (24 and 355 ps in 1,4-dioxane solution) have previously been assigned as the excited-state lifetimes of the enol and diketo species of curcumin by considering the presence of diketo form (<5%) in solution [5,12,13,17]. However, the formation of the triplet state with the 210 ps time constant from the singlet state of curcumin in the enol form is considered, based on the observation of the ESA signals at 765 nm. A weak ESA band of the triplet state has also been observed in the transient absorption results in 1,4-dioxane solution [5,12].

On the other hand, the excited-state dynamics of curcumin in a strong hydrogen-bond acceptor, DMSO, appeared more complicated with the ultrafast state transition in 1–2 ps. The ESA band (595 nm) and the SE band (~460 nm) of the LE state convert rapidly to the ICT state, of which the ESA band appears at 495 nm and the SE band at 660 nm. The global analysis of these kinetic traces showed four kinetic components of 0.11 ± 0.01, 1.2 ± 0.1, 5.8 ± 0.3, and 178 ± 4 ps. The fastest (0.11 ps) component represents the vibrational relaxation of the LE state, appearing as the decays of the ESA and SE bands originating from the Franck–Condon region. The kinetic components of 1.2 and 5.8 ps commonly observed in both the ESA (495 nm) and SE (660 nm) bands represent the ICT dynamics and the subsequent vibrational relaxation of the ICT state. Lastly, the 178 ps component represents the lifetime of the ICT state. Similarly, a fast ICT (0.8 ± 0.1 ps) and the subsequent vibrational relaxation dynamics (11.4 ± 0.2 ps) of the ICT state were observed from the transient absorption measurements in methanol (strong hydrogen-bond donor and acceptor) solution in addition to the decay (128 ± 1 ps) of the ICT state (see Figure S5 in the Supplementary Materials). Transient absorption results of curcumin in DMSO and methanol solutions appear quite similar to the previous results, where the fast (0.11 ps) vibrational relaxation dynamics of the LE state was not separately measured [5].

The solvation dynamics on the ultrafast time scales of 0.1–0.3 ps have been often observed in many organic solvents from the time-resolved fluorescence measurements [38]. The vibrational relaxation dynamics (0.11 ps) in the LE state of curcumin observed from the transient absorption measurements can be strongly related to the ultrafast solvation dynamics of DMSO. Maroncelli et al. reported the multimodal solvation dynamics of DMSO, including the fastest 0.21 ps component by the time-resolved fluorescence of coumarin dye [38]. However, time-resolved electronic spectroscopy measurements can only provide limited information on the ultrafast solvation dynamics accompanying the excited-state processes. Time-resolved vibrational spectroscopy measurements, including numerous solute and solvent vibrational modes, may provide the key experimental evidence for such ultrafast solvation dynamics in addition to the ICT process of curcumin.

### 2.3. Excited-State Dynamics of Curcumin by FSRS

**Figure 3:**
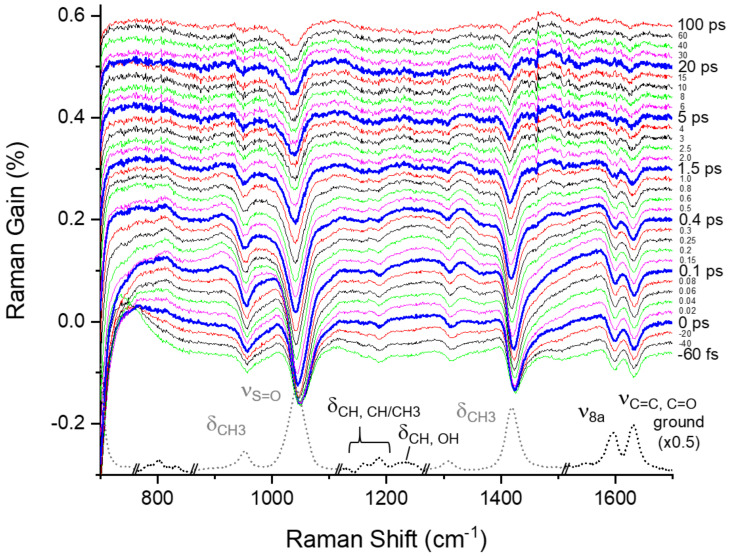
Femtosecond stimulated Raman spectra of curcumin in DMSO obtained with 403 nm excitation. The ground state spectrum taken at −5 ps was subtracted from each spectrum to show the temporal changes clearly. The intensities of the solvent vibrational bands including ν_S=O_ at 1043 cm^−1^ and δ_CH3_ at 952, 1307, and 1419 cm^−1^ were further rescaled (×0.2) in the ground spectrum.

Figure 3 shows the time-resolved stimulated Raman spectra of curcumin obtained with the 403 nm excitation, where the fluorescence backgrounds were removed by low-order polynomial fits, as shown in Figure S6 in the Supplementary Materials. The difference spectra from the ground state spectrum taken at a time delay of −5 ps were displayed with the ground state spectrum. The major vibrational bands of curcumin appear at 1160 and 1186 (δ_O__H, CH/CH3_), 1596 (ν_8a_), and 1631 cm^−1^ (ν_C=C, C=O_) in the ground state spectrum [39,40,41,42]. As shown in Figure S1 in the Supplementary Materials, a couple of weak bands also appear in the ground state spectrum at 981 (ν_C–C_ + δ_CCC_), 1225, 1245, and 1305 (δ_C__H, O__H_), and 1428 cm^−1^ (δ_CH3, CH_), which overlaps the solvent vibrational bands of DMSO partially. The vibrational assignments of the ground-state Raman bands are based on the density functional method simulations, which are described in detail in the Supplementary Materials.

**Figure 4:**
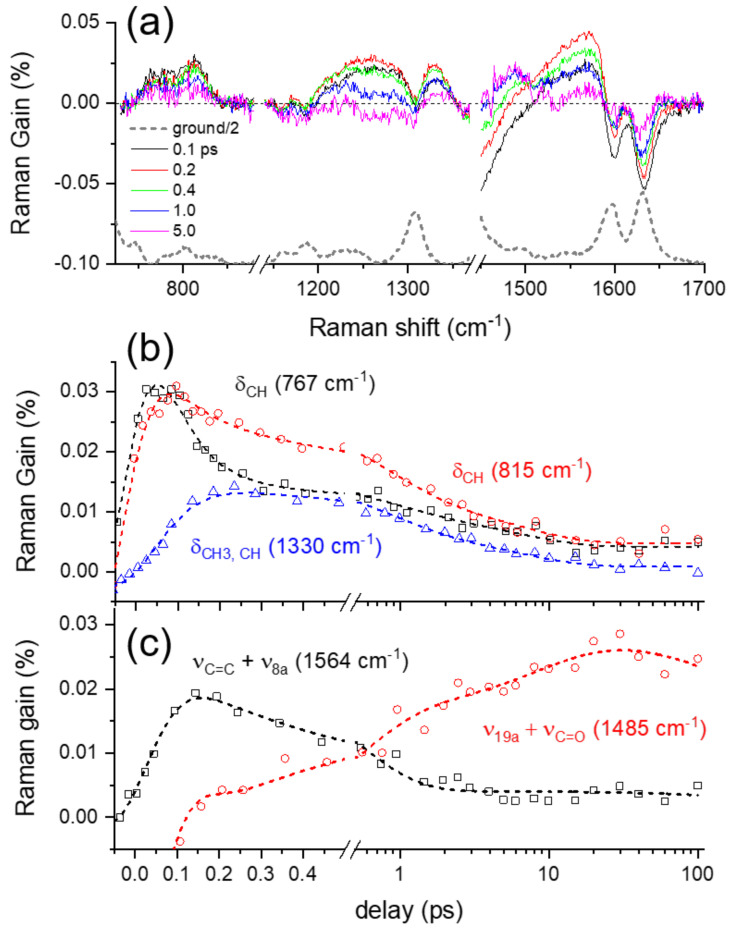
(**a**) Excited-state Raman spectra of curcumin obtained from FSRS measurements with 403 nm excitation and the ground-state Raman spectrum (**b**), (**c**) population dynamics of the major vibrational modes of curcumin in the S_1_/LE and S_1_/CT states. Open symbols represent the experimental data and dotted lines represent the fit results.

Several vibrational bands, including two negative bands at 1601 and 1631 cm^−1^ and weak positive bands at 767, 815, 1254, 1330, 1485, and 1564 cm^−1^, appear in the excited-state Raman spectra of curcumin shown in Figure 4a. The kinetics for the major vibrational modes of curcumin in the excited state are summarized in Figure 4b,c. The detailed kinetic analysis based on the IRF Gaussian-convoluted exponential functions and the dispersive Gaussian functions for the cross-phase modulation (CPM) [33,43] between the actinic pump and Raman probe pulses are described in the Supplementary Materials and summarized in Tables S1 and S2. Several excited-state Raman bands of curcumin, including δ_CH_ at 767 and 815 cm^−1^, δ_CH, OH_ at 1215/1260, δ_CH3, CH_ at 1330, and ν_C=C_ + ν_8a_ at 1564 cm^−1^, represent the vibrational bands of curcumin in the LE state. Another excited-state mode at 1485 cm^−1^ shows distinct kinetics from all the LE vibrational modes, and thus is considered as the ν_19a_ + ν_C=O_ mode of curcumin in the charge-transferred (CT) state. All the excited-state Raman bands of curcumin share common decay or growth dynamics of 0.6–0.8 ps for the ICT process, which is slightly faster than the 1.2 ps observed in the transient absorption measurements. It is also interesting to note that the vibrational relaxation dynamics in the LE potential surface are clearly resolved in the dynamics of the LE vibrational modes. As shown in Figure 4b, the δ_CH_ at 767 cm^−1^ and other LE bands, including δ_CH3, CH_ at 1330 and ν_C=C_ + ν_8a_ at 1564 cm^−1^, show the ultrafast vibrational relaxation dynamics of <60 fs, which was measured slightly slower as 110 fs in the transient absorption measurements. On the other hand, the LE band δ_CH_ at 815 cm^−1^ appears insensitive to the vibrational relaxation in the LE state, which shows that the vibrational relaxations in the LE state occur via specific vibrational modes of curcumin. The subsequent relaxation dynamics of 6–9 ps were also observed in most of the excited-state Raman bands of curcumin, which is then interpreted as the vibrational relaxation in the CT state. While transient absorption measurements provide limited information regarding the excited-state dynamics, including the ICT and vibrational relaxation dynamics occurring on the excited state potential surfaces, more detailed information on the structural changes of curcumin during the ICT process and subsequent vibrational relaxations have been observed in the FSRS measurements via numerous excited-state vibrational modes of curcumin.

### 2.4. Ground-State Vibrational Modes of Curcumin

**Figure 5:**
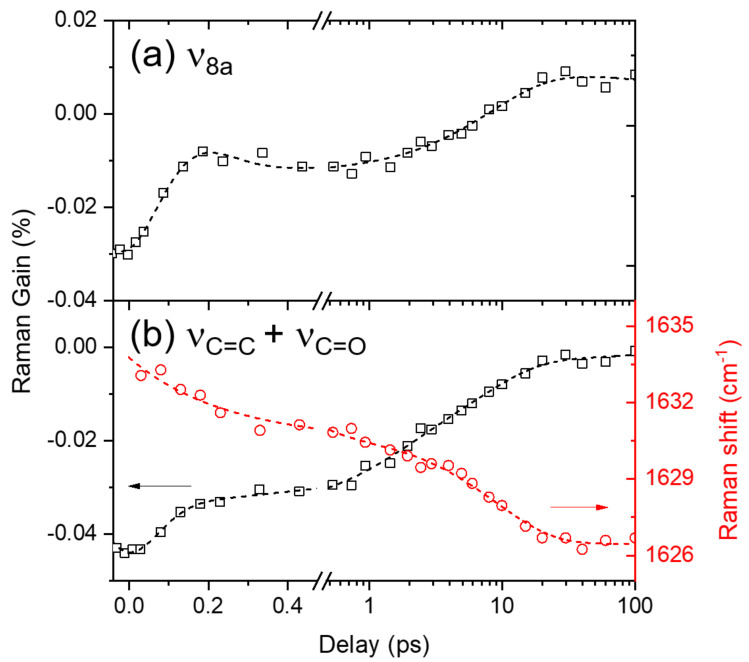
The population dynamics and frequency shifts of the ground-state vibrational modes: (**a**) ν_8a_ and (**b**) ν_C=C, C=O_ of curcumin in DMSO solution obtained from FSRS measurements with 403 nm excitation. Open symbols represent the experimental results and dotted lines represent the fit results.

Moreover, the ground-state vibrational bands of curcumin, ν_8a_ and ν_C=C, C=O_ appear as negative bands centered at 1596 and 1631 cm^−1^, respectively, in the excited-state Raman spectra in Figure 3 and Figure 4a. At first, these negative bands were considered as the ground-state bleaching of curcumin because they appear at approximately similar frequencies as the ground-state vibrational bands. However, the population dynamics of these negative bands shown in Figure 5 appears much faster (8.4 ps for ν_8a_ and 1.3 and 7.1 ps for ν_C=C, C=O_) than the excited-state lifetime (178 ps) of curcumin observed in the transient absorption measurements, which were similarly analyzed by the IRF Gaussian-convoluted exponential functions and the dispersive Gaussian functions for the CPM artifacts. Table S3 in the Supplementary Materials summarizes the fit results. These negative vibrational bands of curcumin are considered strongly related to the changes in the solvation of DMSO rather than the bleaching of the ground-state Raman bands. The recovery dynamics of the ν_8a_ and ν_C=C, C=O_ modes of curcumin with the time constants of 7.1–8.4 ps are quite similar to the vibrational relaxation dynamics in the CT state, while 1.3 ps recovery of ν_C=C, C=O_ is compatible with the ICT dynamics of curcumin. Interestingly, the ICT dynamics of curcumin in the excited state is only observed with the ν_C=C, C=O_ modes. Some excited-state vibrational bands may coexist with the negative Raman bands of ν_C=C, C=O_ modes. However, the spectral changes in the ν_C=C, C=O_ band during the ICT, shown in Figure 4a and Figure S3 in the Supplementary Materials, do not support the co-existence of the excited state Raman bands, which is further confirmed by the strong red-shifts in the ν_C=C, C=O_ band (1633→1626 cm^−1^) during the ICT and the subsequent vibrational relaxation in the CT state. The peak shifts in the ν_C=C, C=O_ band were fit to a bi-exponential function with the time constants of 0.23 and 9.0 ps.

The solvent DMSO is known as an effective hydrogen bond acceptor, so the strong solvation on curcumin via hydrogen bonding between the hydroxyl groups of curcumin and the sulfoxide group of DMSO would be expected [44,45,46]. Figure S3 in the Supplementary Materials compares the negative Raman bands from FSRS measurements with the ground-state Raman spectra of curcumin in various polar and nonpolar solvents, in the frequency region of ν_8a_ and ν_C=C, C=O_ modes. The ν_8a_ bands, which consist of multiple vibrational normal modes, may be combined or separated depending on the solvent. On the other hand, the ν_C=C, C=O_ bands appear more sensitive to solvent interactions. The red-shifts (1636→1631 cm^−1^) in the ν_C=C, C=O_ bands appear directly related to the solvent interaction via hydrogen bonding. Nibbering et al. investigated the hydrogen-bonding dynamics of coumarin 102 in chloroform and phenol, where the ultrafast hydrogen bond cleavages (shorter than 200 fs) were observed in the frequency shifts of ν_C=O_ of coumarin 102 and ν_OH_ of phenol [47,48]. Similarly, the ultrafast dynamics of 0.23 ps in the negative ground-state ν_C=C, C=O_ modes of curcumin is considered to originate from the instantaneous changes in the solvation of DMSO molecules around the solute curcumin. The solvation dynamics of DMSO has been observed in the fluorescence upconversion study of a coumarin dye, where the multimodal relaxation dynamics including the fastest (0.21 ps) component are observed [38]. Thus the 0.23 ps dynamics observed from the ground-state ν_C=C, C=O_ modes of curcumin is considered to originate from the solvation dynamics of DMSO. Although we have not observed such solvation changes from the excited-state vibrational modes of curcumin, the ultrafast changes in the solvation shells of DMSO, including the breakage of the hydrogen bonding with curcumin or intermolecular interactions between DMSO molecules, have been indirectly probed by the neighbor curcumin molecules in the ground-state via the peak-shifts of the ν_C=C, C=O_ Raman bands. Similarly, the slower 7–9 ps dynamics in both the ν_8a_ and ν_C=C, C=O_ modes of curcumin can be interpreted as originating from the vibrational relaxation in the CT state.

### 2.5. Solvation Dynamics of DMSO

**Figure 6:**
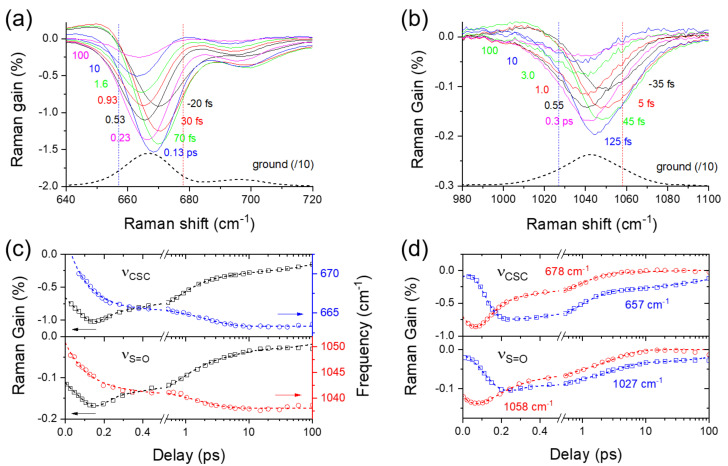
Transient spectral changes in the (**a**) ν_CSC_ and (**b**) ν_S=O_ of solvent DMSO obtained from FSRS measurements of curcumin with 403 nm excitation. The ground-state spectra of DMSO were also compared. (**c**) The population dynamics and frequency shifts of ν_CSC_ and ν_S=O_ modes of solvent DMSO, (**d**) the population dynamics of the sub-bands of ν_CSC_ and ν_S=O_ modes of solvent DMSO. Open symbols represent the experimental data and dotted lines represent the fit results.

The solvation dynamics of DMSO before and after the ICT of curcumin in the excited state can also be observed directly from the solvent vibrational mode of DMSO due to the wide spectral coverage of FSRS. Ultrafast solvation dynamics of DMSO have been first reported by our recent studies of excited-state intramolecular proton transfer of 1,2-dihydroxyanthraquinone, where the ultrafast population dynamics of 110 and 170 fs between the “free” and “aggregated” species of DMSO has been used for the observation of the ultrafast proton transfer dynamics [33,34]. Figure 6 shows the ν_CSC_ and ν_S=O_ modes of DMSO observed in the FSRS of curcumin with 403 nm excitation. To avoid unwanted effects of the fluorescence signals, the fluorescence backgrounds in the ν_CSC_ and ν_S=O_ bands of DMSO were removed by linear backgrounds in a shorter range (see Figure S7 in the Supplementary Materials). The ν_S=O_ appears at 1043 cm^−1^ in Figure 6b as an asymmetric band, which consists of multiple sub-bands representing a monomer (free), dimer (aggregated), or hydrogen-bonded species of DMSO, depending on the intermolecular interactions between DMSO molecules [33,34,44,49,50,51]. The ν_CSC_ bands appear at 667 and 697 cm^−1^ in Figure 6a as the symmetric and antisymmetric stretching, respectively. Similarly, the symmetric ν_CSC_ band centered at 667 cm^−1^ consists of the sub-bands of free, aggregated, and hydrogen-bonded species of DMSO [33,44]. Furthermore, the δ_CH3_ mode appears as a major band at 1419 cm^−1^ in addition to other rocking or deformation bands at 952 and 1307 cm^−1^ [49].

The spectral changes in the solvent vibrational modes of DMSO are described mainly by the dispersive signals around the zero time delay, and the negative signals which last much longer (100 ps or longer) after the excitation, which is considered as vibrationally hot ground-state bands created by the excited-state deactivations of chromophores [33]. Figure 6c compares temporal changes in the intensities and center frequencies of the ν_CSC_ and ν_S=O_ modes upon the photoexcitation of curcumin. The population changes of both vibrational modes were evaluated with a bandwidth of 25 cm^−1^ not to include any inter-band spectral changes. The vibrational intensities of the ν_CSC_ and ν_S=O_ modes show multiple exponential decays of 0.72, 4.4, and 163 ps, which is consistent with the ICT dynamics, the vibrational relaxation in the CT state, and the population decay of the CT state of curcumin in DMSO. It appears that the solvent vibrational modes of DMSO are strongly affected by the excited-state dynamics of a chromophore when the chromophore is strongly solvated by DMSO molecules by hydrogen bonding. The perturbation of the solvent vibrational modes of DMSO in curcumin solution represents the transient changes in the solvation shells, including hydrogen bonding with the solute curcumin and intermolecular interaction between DMSO molecules [5,33]. The frequency shifts in the ν_CSC_ and ν_S=O_ modes of DMSO with the ICT of curcumin, shown in Figure 6c, are also considered as the evidence of ultrafast solvation changes of DMSO. From the kinetic analysis based on the biexponential functions, two fast time constants of 0.11 and 1.8 ps were retrieved for the vibrational Stokes shifts of DMSO. The instantaneous changes in the solvation shells, such as intermolecular hydrogen bonding between the solute and solvent molecules, have been reported in polar protic solvents [27,52,53,54,55]. Scholes et al. reported the inertial solvent responses of water and methanol in the ultrafast time scales of 40–150 fs in the light-harvesting proteins [54]. The coherent oscillations in the vibrational modes of chromophores in protic and aprotic polar solvents have often been interpreted as the solvation dynamics of water or DMSO with the time constants of 190–250 fs, which is considered to facilitate the ultrafast proton and charge transfers in the excited states [27,55].

Furthermore, the ultrafast changes of the ν_CSC_ and ν_S=O_ modes in time-resolved Raman spectra of DMSO have been compared between the sub-bands for the “free”, “aggregated”, and “hydrogen-bonded” species of DMSO. The frequency differences of the ν_CSC_ and ν_S=O_ modes of DMSO between the “free”, “aggregated”, and “hydrogen-bonded” species have been confirmed by the molecular dynamics simulations and infrared and Raman measurements in numerous polar and nonpolar solvents [33,44]. Figure 6d compares the dynamics of the ν_CSC_ modes between the sub-bands at 657 and 678 cm^−1^, representing the free and hydrogen-bonded species, respectively. The dynamics of the ν_S=O_ modes between the sub-bands at 1058 and 1027 cm^−1^, representing the free and hydrogen-bonded species, respectively, were also compared. The ultrafast reversal in the Raman intensities of the free and hydrogen-bonded species of DMSO, in <0.2 ps, is seen in both the ν_CSC_ and ν_S=O_ modes of DMSO. Because the transient Raman bands of DMSO show dependence on the excited-state dynamics of curcumin with three time constants of 0.72, 4.4, and 163 ps (Figure 6c), the dynamics of both sub-bands of the ν_CSC_ and ν_S=O_ modes were fit to the Gaussian-convoluted exponential functions for the ultrafast inter-sub-band reversal in addition to three Gaussian-convoluted exponentials with fixed time constants for the excited-state dynamics of curcumin (from Figure 6c). As summarized in Table S5 in the Supplementary Materials, the ν_CSC_ and ν_S=O_ modes show the ultrafast reversals of 20–50 fs between the free and hydrogen-bonded species.

Moreover, the inter-sub-band conversions of the ν_CSC_ and ν_S=O_ modes occur oppositely; the free sub-band (at 657 cm^−1^) of the ν_CSC_ converts to the hydrogen-bonded sub-band (at 678 cm^−1^) in the ultrafast time scales of 20–50 fs while the hydrogen-bonded sub-band (at 1027 cm^−1^) of the ν_S=O_ converts to the free sub-band (at 1058 cm^−1^) at the same time. The mode-specific changes in the Raman spectrum of the solvent DMSO are considered as the key experimental results for the structural changes of DMSO molecules in the solvation shells during the ICT of curcumin in the excited state. The ultrafast (20–50 fs) decrease of the hydrogen-bonded species for the ν_S=O_ mode seems to be inconsistent with the increase of the hydrogen-bonded species for the ν_CSC_ mode occurring at the same time. However, all DMSO molecules in the solvation shells or near the photoexcited curcumin molecules can be responsible for the transient Raman spectra of DMSO observed with the ICT of curcumin. The solvation of DMSO with curcumin is expected to occur mainly via the hydrogen bond interactions between the sulfoxide group and the hydroxyl groups of curcumin. Thus, the decrease in the hydrogen-bonded species of the ν_S=O_ mode may represent the hydrogen bond disruptions between the hydroxyl and sulfoxide groups, while the increase in the hydrogen-bonded species of the ν_CSC_ mode represents the resulting changes in the intermolecular interactions between DMSO molecules in the solvation shells. The experimental evidence for the ultrafast changes in the solvation shells of DMSO obtained from FSRS measurements of curcumin requires further verification by theoretical investigations, including molecular dynamics simulations. However, such molecular dynamics simulations may require the non-adiabatic quantum mechanical treatments of the chromophore in addition to the classical molecular dynamics, where the computing resources severely limit the size of the chromophores and the accuracy of simulation results [56,57]. Further experimental explorations with the binary mixtures of DMSO and water, or DMSO and nonpolar solvents, may confirm the proposed ultrafast changes of DMSO molecules in the solvation shells accompanying the excited-state processes of chromophores with strong solvent interactions via hydrogen bonding.

## 3. Materials and Methods

### 3.1. Chemicals

Curcumin (TCI, Tokyo, Japan) and all solvents, including chloroform and DMSO, were used without further purification. Curcumin solution of 2 mM in DMSO was used for time-resolved stimulated Raman measurements. The sample was recirculated in a 0.5 mm-thick quartz flow cell (Starna Scientific, Ilford, UK) by a peristaltic pump to minimize photodamage. Dilute solutions of 0.2 mM in chloroform and DMSO were used for transient absorption measurements in a 2.0 mm-thick quartz cell stirred by a small bar magnet. All the spectral measurements were carried out at room temperature.

### 3.2. Femtosecond Transient Absorption Setup

A homebuilt transient absorption setup based on a 1 kHz Ti:Sapphire regenerative amplifier was used for transient absorption measurements [58,59]. The broadband (450–1000 nm) probe pulses were generated by focusing fundamental pulses in a sapphire window (3 mm thick; EKSMA optics, Vilnius, Lithuania) and filtered by a short pass filter (700CFSP; Omega Optical, Brattleboro, VT, USA). The actinic pump pulses of 403 nm were generated by the sum-harmonic generation of the fundamental pulses and compressed by a prism pair compressor (SF10; Edmund Optics, Barrington, NJ, USA). Pulse energy of 150 nJ was used for the actinic pump.

### 3.3. Femtosecond Stimulated Raman Spectroscopy (FSRS) Setup

The details of a homebuilt Raman setup used for the stimulated Raman measurements are described elsewhere [29,55]. The stimulated Raman probe consists of the broadband (850–1000 nm) femtosecond Raman probe pulses generated by the supercontinuum generation in a YAG window (4 mm thick; Newlight Photonics, North York, ON, Canada), and the narrowband picosecond Raman pump pulses generated by a home-built grating filter (1200 gr/mm). The actinic pump pulses at 403 nm were produced by the sum-harmonic generation of the fundamental and compressed by a pair of chirped mirrors (−25 ± 10 fs^2^ group delay dispersion; Layertec GmbH, Mellingen, Germany). The pulse energies of the Raman pump and actinic pump were set at 550 and 250 nJ, respectively, at the sample position. The probe pulses through the sample were dispersed in an f = 320 mm spectrograph and collected in a fast CCD detector (PIXIS 100; Princeton Instruments, Trenton, NJ, USA). The IRF of FSRS measurements was obtained from the transient absorption background signals in the Raman probe recorded without the Raman pump pulses, which was used for the chirp correction for the FSRS results.

## 4. Conclusions

The ultrafast ICT dynamics of curcumin in DMSO solution have been investigated by FSRS, where strong solvent interactions via hydrogen bonding exist. Due to the high spectral and temporal resolutions of FSRS, the multi-faceted excited-state dynamics of curcumin, including the ultrafast ICT dynamics (0.6–0.8 ps) and the vibrational relaxation in the CT state (6–9 ps), were observed from numerous solute and solvent Raman bands of curcumin in DMSO, in addition to the population dynamics (173 ps). Interestingly, the bleaching-like negative ground-state Raman bands of curcumin in the ν_8a_ and ν_C=C, C=O_ show even faster dynamics of 0.23 ps, which is interpreted as the vibrational relaxation in the LE state. All the excited-state dynamics of curcumin in DMSO were also probed by the solvent Raman bands of DMSO, the ν_CSC_ and ν_S=O_. Separate from nonlinear dispersive CPM artifacts, the ultrafast (20–50 fs) structural changes of DMSO molecules were observed from the sub-bands of both Raman bands. The solvation dynamics of DMSO have been reported as multimodal. We report one of the fastest solvation dynamics of DMSO with hydrogen bond disruption between the photo-excited curcumin and solvating DMSO molecules and subsequent changes in the intermolecular interactions between DMSO molecules in the solvation shells, which have been proposed from the population dynamics of the ν_CSC_ and ν_S=O_ modes between the “free” and “hydrogen-bonded” species of DMSO. However, further confirmations of the proposed solvation changes are required by FSRS measurements with the binary mixtures of solvents and the theoretical developments, including non-adiabatic quantum mechanical and classical molecular dynamics simulations.

## Supplementary Materials

The following supporting information can be downloaded at: https://www.mdpi.com/article/10.3390/ijms23031727/s1.
