# Supplementary material for: Intramolecular Charge Transfer of Curcumin and Solvation Dynamics of DMSO Probed by Time-Resolved Raman Spectroscopy
Source: Int J Mol Sci. 2022 Feb 2;23(3):1727. doi: 10.3390/ijms23031727 (PMC8835799; doi:10.3390/ijms23031727)
Supplement: Supplementary file 1 [file ijms-23-01727-s001.zip › ijms-1538852-supplementary.pdf]

[Supplementary Materials]

Intramolecular Charge Transfer of Curcumin and Solvation Dynamics of DMSO Probed by Time-Resolved Raman Spectroscopy

Myungsam Jen, Sebok Lee, Gisang Lee, Daedu Lee, and Yoonsoo Pang*

Department of Chemistry, Gwangju Institute of Science and Technology, 123 Cheomdangwagi-ro, Buk-gu Gwangju 61005, Republic of Korea

Table of Contents

1. Ground-state Raman spectra of curcumin and vibrational assignments 2

2. Transient absorption results of curcumin in 1,4-dioxane and methanol 7

3. Background subtraction of time-resolved Raman data for kinetic analysis 9

4. Kinetic analysis of ground- and excited-state Raman bands 11

*Author to whom correspondence should be addressed. E-mail address: ypang@gist.ac.kr.

1. Ground-state Raman spectra of curcumin and vibrational assignments

Figure 1 show the ground-state Raman spectra of curcumin in polar solvent of chloroform, methanol, and dimethyl sulfoxide (DMSO).

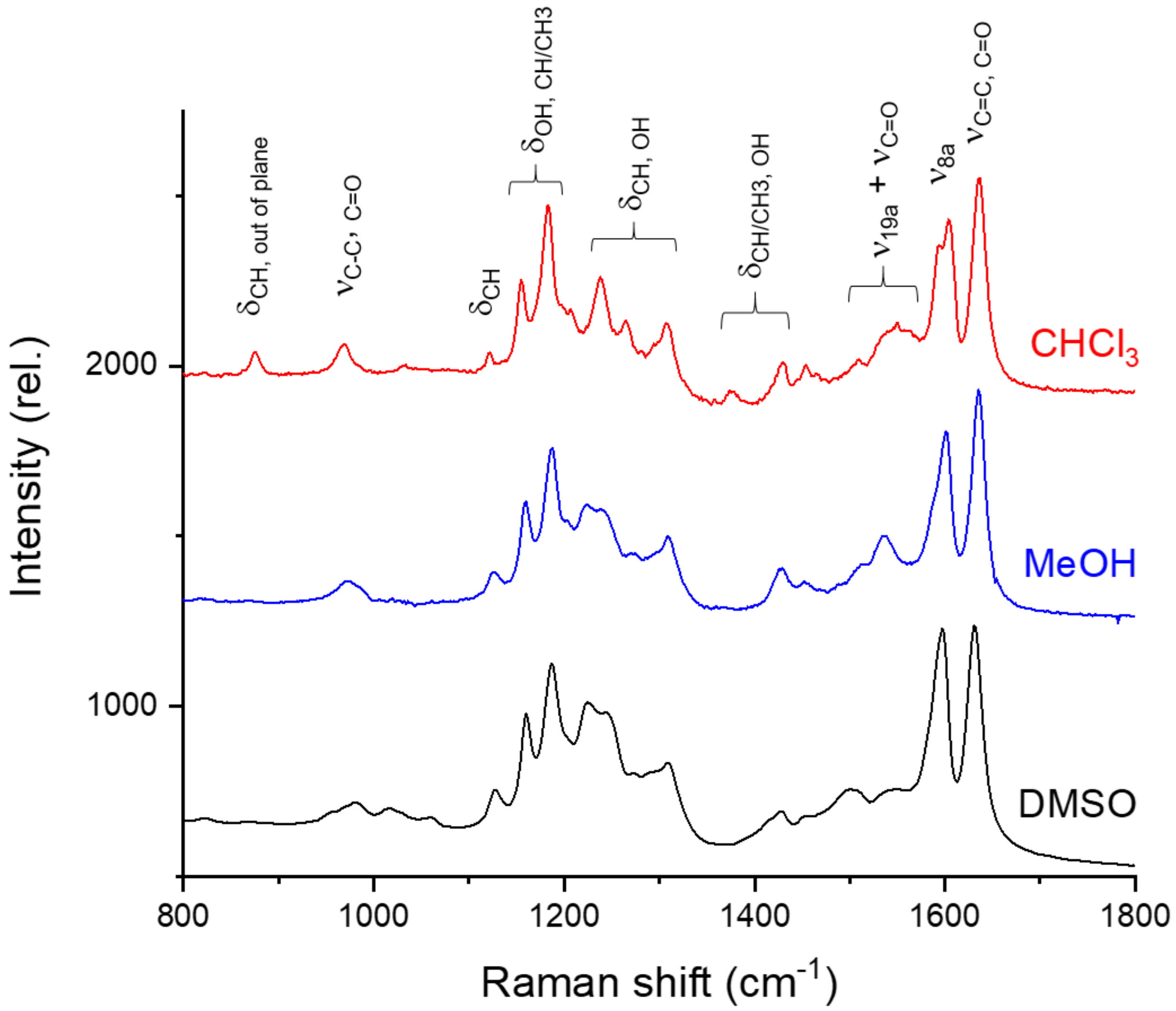

Figure S1. Ground-state Raman spectra of curcumin in $CHCl_3$, methanol, and DMSO solutions obtained with 785 nm conventional Raman setup. The solvent peaks were removed by subtracting the solvent spectra. The vibrational assignments for the major vibrational bands of curcumin was also displayed.

The vibrational assignments of the major ground-state vibrational bands of curcumin were based on the density functional theory (DFT) simulations at B3LYP/6-311G(d,p) level with the polarizable continuum model of DMSO. The Raman spectrum of curcumin in a DMSO solution was obtained with the optimized structure of curcumin in the ground state. The Gaussian 09 software[1] was used for all DFT simulations and all the vibrational frequencies were rescaled with a scaling factor of 0.967.[2, 3] Figure S2 shows the major vibrational normal modes of curcumin in the ground state in DMSO solution.

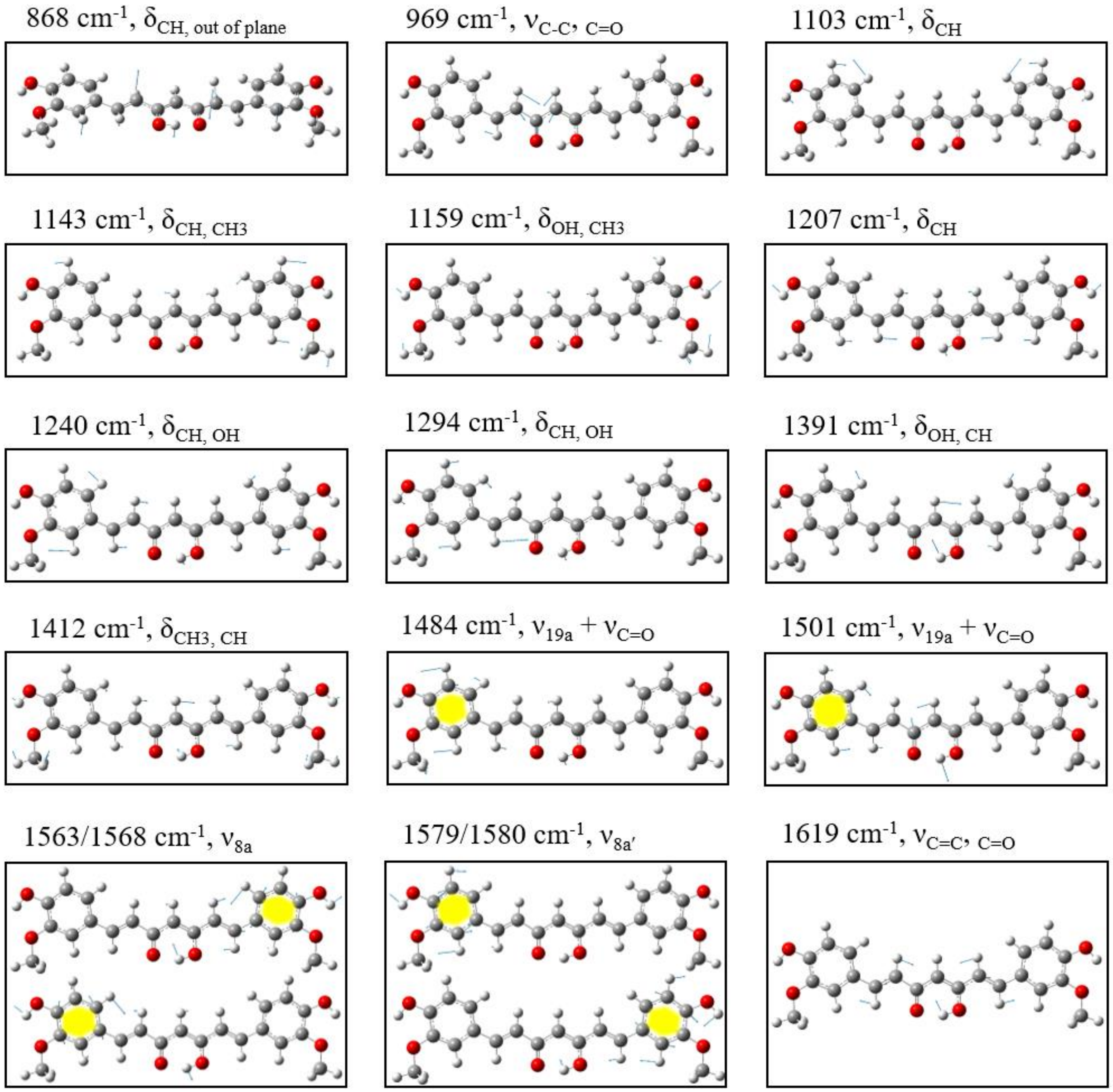

Figure S2. Vibrational normal modes of curcumin in the ground state obtained from the DFT simulations at the B3LYP/6-311G(d,p) level with PCM-DMSO. The vibrational frequencies represent the experimental values.

Figure S3 compares the ν_{8a} and $\nu_{C=C,\ C=O}$ bands of curcumin in the ground state dissolved in several solvents of varying polarity. The ν_{8a} band of curcumin consists of the corresponding modes of one phenyl ring and the other, which appears as the combined asymmetric bands in polar solvents DMSO and methanol and is separated into two vibrational modes in less polar solvents of 1,4-dioxane, benzene, and chloroform. The $\nu_{C=C,\ C=O}$ bands of curcumin appear as the broad combined bands in all the solvents while the $\nu_{C=C,\ C=O}$ bands appearing at 1631 cm^{-1} in DMSO are blue-shifted to 1636 cm^{-1} in chloroform. The negative signals in the ν_{8a} bands of curcumin appear at 1599-1600 cm^{-1}, which is slightly blue-shifted from the ground state maximum position (1597 cm^{-1}). The negative ν_{8a} bands of curcumin do not show considerable peak shifts with the decay although the excited-state Raman band appearing at 1599 cm^{-1} shows a spectral overlap with the negative ν_{8a} bands. On the other hand, the negative $\nu_{C=C,\ C=O}$ bands appearing at 1633 cm^{-1} at early time delays are red-shifted to 1626 cm^{-1} with the decay.

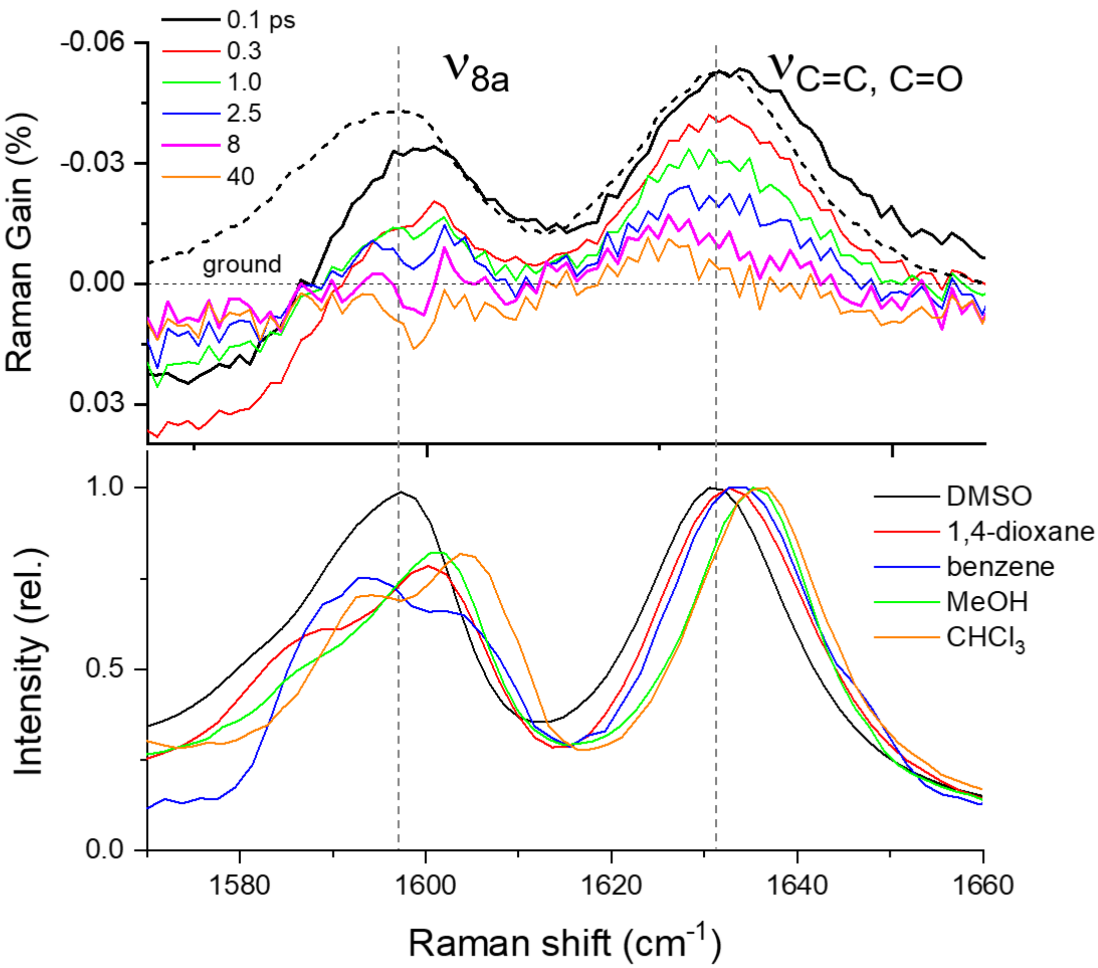

Figure S3. Ground-state Raman spectra of curcumin in the frequency range for ν_{8a} and $\nu_{C=C,\ C=O}$ modes (bottom) in DMSO, 1.4-dioxane, toluene, and chloroform solutions were compared with time-resolved Raman spectra (top) from FSRS measurements with 403 nm excitation. The solvent peaks were removed by subtracting the solvent spectra. The intensities of the time-resolved Raman spectra (top) were inversed for easy comparison.

2. Transient absorption results of curcumin in 1,4-dioxane and methanol

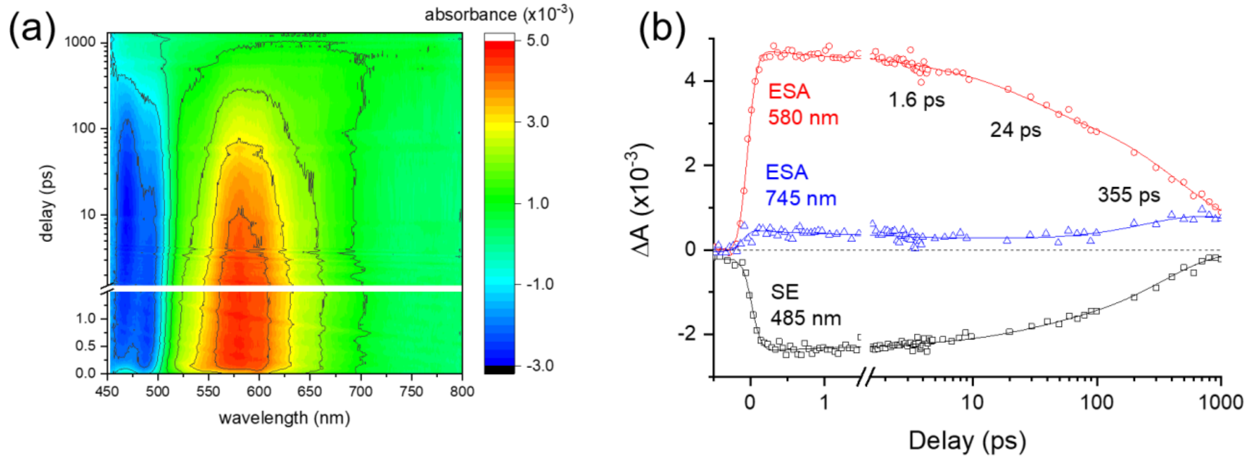

Figure S4. Transient absorption results of curcumin in 1,4-dioxane obtained with 403 nm excitation; (a) surface plot, (b) kinetics of the ESA band (580 nm) and SE band (485 nm) of the LE state, and the ESA band (785 nm) of the triplet state.

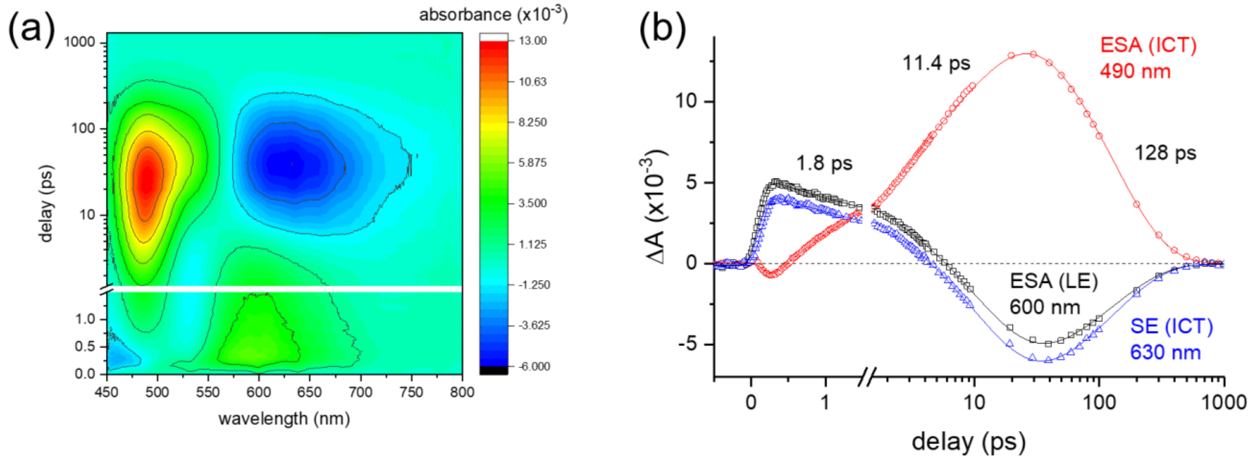

Figure S5. Transient absorption results of curcumin in methanol obtained with 403 nm excitation; (a) surface plot, (b) kinetics of the ESA band of the LE state (600 nm), ESA band (490 nm) and SE band (630 nm) of the ICT state.

3. Background subtraction of time-resolved Raman data for kinetic analysis

The fluorescence backgrounds in the FSRS results were removed by the low-order polynomial fit lines connecting several points of 570, 860, 1142, 1360, 1700 cm^{-1}, where no apparent Raman band does not exist. Figure S4(a) shows the representative polynomial fit lines at time delays of 0.1, 1, 2, 5, and 100 ps, and the resulting stimulated Raman spectrum of curcumin in DMSO at each time delay was shown in Figure S4(b).

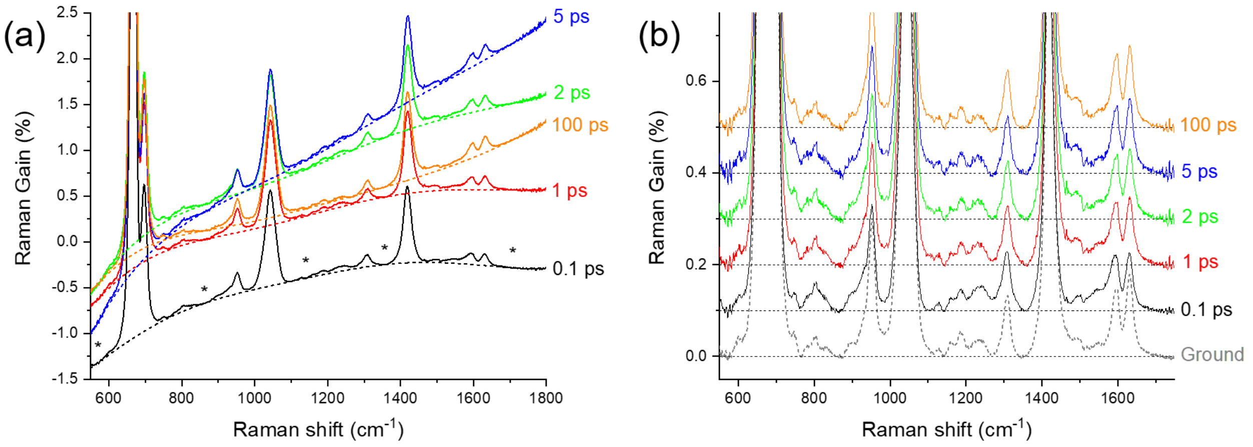

Figure S6. The fluorescence backgrounds in (a) transient Raman spectra of curcumin in DMSO were removed with the polynomial fit functions through several points at 570, 860, 1142, 1360, and 1700 cm^{-1}, (b) transient Raman spectra without the fluorescence backgrounds.

The difference FSRS spectra obtained with the polynomial background lines, shown in Figure 3, may contain the fluctuating signals originating from the fluorescence signals. Especially, the polynomial fluorescence backgrounds in the frequency ranges for the strong ν_{CSC} and $\nu_{S=O}$ modes of DMSO appear inappropriate for the accurate analysis of the ultrafast solvation dynamics upon the ICT of curcumin. Instead, the background signals for these vibrational modes were further determined using the linear functions in the narrower frequency ranges containing these vibrational modes of interest than the polynomial functions covering all the frequency ranges. Figure S7 shows examples of the linear backgrounds for the ν_{CSC} and $\nu_{S=O}$ modes of DMSO, which shows clear difference from the polynomial background lines.

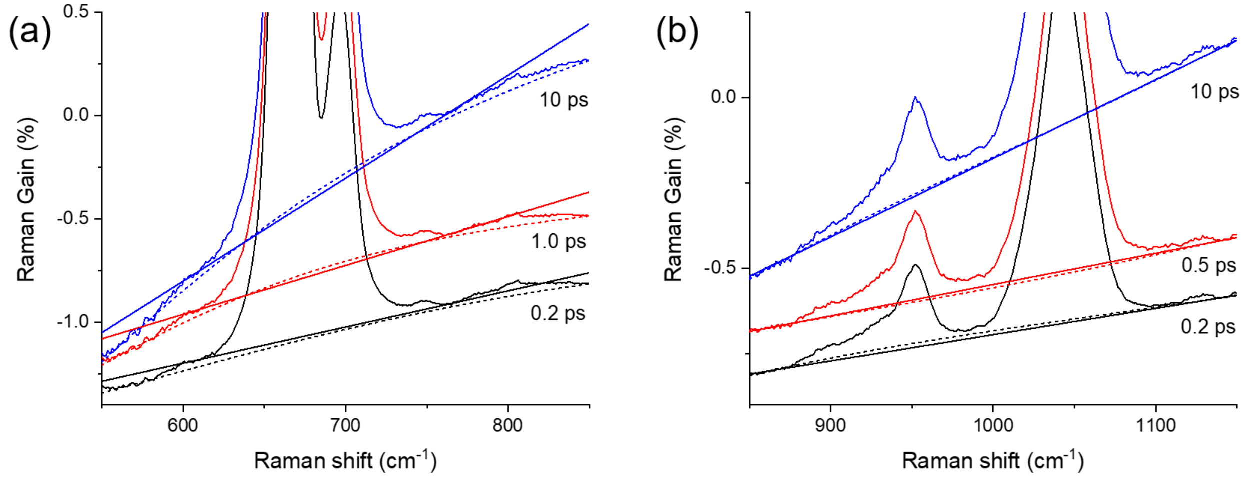

Figure S7. The fluorescence background of the transient Raman spectra of curcumin in DMSO in (a) the ν_{CSC} and (b) $\nu_{S=O}$ modes of DMSO were removed with the linear functions over a wide frequency ranges. The linear (solid lines) and polynomial (dotted lines) backgrounds at several time delays were compared.

4. Kinetic analysis of ground- and excited-state Raman bands

Transient Raman bands of the δ_{CH} modes at 767 and 815 cm^{-1} and $\delta_{CH3,\,CH}$ mode at 1330 cm^{-1} of curcumin, shown in Figure 4(b), were fit with the instrument response function (IRF) Gaussian-convoluted exponential functions,

$$\mathrm{GE}_i\left(t\right) = A_i \exp\left(\frac{\omega^2}{2\tau_i^2} - \frac{t-t_0}{\tau_i}\right)\left[1 - erf\left(\frac{\omega^2 - \tau_i(t-t_0)}{\sqrt{2}\omega\tau_i}\right)\right] \tag{S1}$$

where A_i represents amplitude, τ_i exponential lifetime, and ω the width (variance) of a Gaussian function. The excited-state dynamics of the δ_{CH} and $\delta_{CH3,\,CH}$ modes were analyzed in the global fit, where all the lifetimes τ_i, the Gaussian variance ω, and the time zero t_0 were shared over all the species. The fit results displayed in Figure 4(b) were summarized in Table S1.

Table S1. Excited-state dynamics of the δ_{CH} and $\delta_{CH3,\,CH}$ modes of curcumin in FSRS results

shared parameters (ps)					
ω	t_0	τ_1	τ_2	τ_3	τ_4
0.067 ± 0.014	-0.034 ± 0.016	0.064 ± 0.017	0.79 ± 0.19	6.2 ± 1.9	inf. [a]

individual parameters				
vibrational mode	A_1	A_2	A_3	A_4
δ_{CH} (767 cm^{-1})	0.037 ± 0.018	0.003 ± 0.001	0.003 ± 0.001	0.016 ± 0.010
δ_{CH} (815 cm^{-1})	0.014 ± 0.010	0.008 ± 0.001	0.004 ± 0.001	0.017 ± 0.008
$\delta_{CH3,\,CH}$ (1330 cm^{-1})	-0.018 ± 0.006	0.005 ± 0.001	0.003 ± 0.001	0.001 ± 0.001

[a] The ICT lifetime is too long to be determined from FSRS results.

Transient Raman bands of the $\nu_{C=C} + \nu_{8a}$ at 1564 cm^{-1} and $\nu_{8a} + \delta_{CH}$ at 1485 cm^{-1} of curcumin were also fit similarly with the Gaussian-convoluted exponential functions. The strong dispersive pattern in the kinetics of $\nu_{8a} + \delta_{CH}$ mode originating from the cross-phase modulation was additionally fit with an additional sinusoidal exponential function,[4, 5]

$$\begin{aligned} \mathrm{GE}(t) = & \frac{A_d}{\omega_d\sqrt{\pi/2}} \exp\left(-\frac{2(t-t_0)^2}{\omega_d^2}\right) \sin 2\pi f\,(t-t_0+\phi_d) \\ & + \sum_i A_i \exp\left(\frac{\omega^2}{2\tau_i^2} - \frac{t-t_0}{\tau_i}\right)\left[1 - erf\left(\frac{\omega^2 - \tau_i(t-t_0)}{\sqrt{2}\omega\tau_i}\right)\right] \end{aligned} \quad \text{(S2)}$$

where A_d represents the amplitude and ω_d the variance (width) of the Gaussian function, f the oscillation frequency and ϕ_d the phase of the oscillation for the dispersive signal from the cross-phase modulation. The fit results displayed in Figure 4(c) were summarized in Table S2.

Table S2. Excited-state kinetics of the $\nu_{C=C} + \nu_{8a}$ and $\nu_{19a} + \delta_{CH}$ modes of curcumin in FSRS results

shared parameters (ps)					
ω	t_0	τ_1	τ_2	τ_3	τ_4
0.067 ± 0	-0.034 ± 0	0.03 ± 0.02	0.49 ± 0.11	9.0 ± 5.4	602 ± 619

individual parameters [a]								
vibrational mode	A_1	A_2	A_3	A_4	A_d	ω_d (ps)	f (ps^{-1})	ϕ_d (ps)
$\nu_{C=C} + \nu_{8a}$ (1564 cm^{-1})	-41 ± 16	11 ± 2	-	2 ± 0	-	-	-	-
$\nu_{19a} + \delta_{CH}$ (1485 cm^{-1})	-	-11 ± 2	-6 ± 2	14 ± 2	-8 ± 1	0.20 ± 0.03	2.4 ± 0.3	0.05 ± 0.00

[a] All the amplitudes A_i and A_d are displayed in thousandths.

The ground-state Raman bands of curcumin, the ν_{8a} at 1601 cm^{-1} and $\nu_{C=C, C=O}$ at 1631 cm^{-1} were also fit with the sum of the Gaussian-convoluted exponential functions and the sinusoidal exponential functions for the CPM signals. The fit results displayed in Figure 5 were summarized in Table S3. The peak shifts of the $\nu_{C=C, C=O}$ mode of curcumin were also fit with a bi-exponential functions, where the fast (0.23 ± 0.05 ps, 45%) and slow (9.0 ± 1.1 ps, 55%) components were found.

Table S3. Photoinduced kinetics of the (ground-state) ν_{8a} and $\nu_{C=C, C=O}$ modes of curcumin in FSRS results

shared parameters (ps)						
ω	t_0					
0.067 ± 0	-0.044 ± 0.037					
individual parameters [a]						
vibrational mode	A_1	τ_1 (ps)	A_2	τ_2 (ps)	A_3	τ_3 (ns)
ν_{8a} (1601 cm^{-1})	-	-	-11 ± 1	8.4 ± 1.2	4 ± 1	0.6 ± 1.3
$\nu_{C=C, C=O}$ (1631 cm^{-1})	-6 ± 3	1.3 ± 0.8	-10 ± 3	7.1 ± 2.9	-1 ± 1	0.17 ± 0.35
	A_d	ω_d (ps)	f (ps^{-1})	ϕ_d (ps)		
ν_{8a} (1601 cm^{-1})	-9 ± 2	0.31 ± 0.05	1.5 ± 0.1	0.84 ± 0.10		
$\nu_{C=C, C=O}$ (1631 cm^{-1})	-70 ± inf.	0.22 ± 0.04	0.06 ± 10.7	-0.25 ± 0.16		

[a] All the amplitudes A_i and A_d are displayed in thousandths.

The solvent Raman bands of DMSO, the ν_{CSC} (centered at 667 cm^{-1}) and $\nu_{S=O}$ modes (centered at 1043 cm^{-1}) show ultrafast changes with the ICT of curcumin in the excited state. First, the population changes in the ν_{CSC} and $\nu_{S=O}$ modes were evaluated at each peak center with a wide bandwidth of 25 cm^{-1} not to include any inter-band changes. Due to strong CPM signals contained in both the solvent vibrational modes, the kinetics of the ν_{CSC} and $\nu_{S=O}$ modes were fit with the sum of the Gaussian-convoluted exponential functions and the sinusoidal exponential functions for the CPM signals. The peak shifts of the ν_{CSC} and $\nu_{S=O}$ modes of DMSO were also fit with a bi-exponential functions. The fit results for the population changes and peak shifts of the ν_{CSC} and $\nu_{S=O}$ modes displayed in Figure 6(c) were summarized in Table S4.

The population changes of the ν_{CSC} and $\nu_{S=O}$ modes between the sub-bands such as "free", "aggregated", and "hydrogen-bonded" species of DMSO were evaluated at 657 and 678 cm^{-1} for the ν_{CSC} mode, and at 1027 and 1058 cm^{-1} for the $\nu_{S=O}$ mode, as shown in Figure 6(d). The kinetics of both sub-bands in the ν_{CSC} and $\nu_{S=O}$ modes of DMSO were also fit with the sum of the Gaussian-convoluted exponential functions and the sinusoidal exponential functions for the CPM signals. As shown in Figure 6(d), both sub-bands show population transfers in 0.2 ps

Table S4. Solvation dynamics of DMSO in the ν_{CSC} and $\nu_{S=O}$ modes observed in FSRS of curcumin

(a) Population dynamics

shared parameters (ps)				
ω [a]	t_0 [a]	τ_1	τ_2	τ_3
0.040 ± 0.002	-0.038 ± 0.002	0.72 ± 0.02	4.44 ± 0.43	163 ± 7

individual parameters				
vibrational mode	A_1	A_2	A_3	
ν_{CSC} (667 cm^{-1})	-0.349 ± 0.005	-0.078 ± 0.005	-0.141 ± 0.002	
$\nu_{S=O}$ (1043 cm^{-1})	-0.053 ± 0.002	-0.023 ± 0.002	-0.017 ± 0.001	
	A_d	ω_d (ps)	f (ps^{-1})	ϕ_d (ps)
ν_{CSC} (667 cm^{-1})	-0.146 ± 0.017	0.295 ± 0.012	3.07 ± 0.06	-0.06 ± 0.02
$\nu_{S=O}$ (1043 cm^{-1})	-0.022 ± 0.002	0.326 ± 0.031	3.06 ± 0.10	-0.71 ± 0.02

(b) Frequency shifts

vibrational mode	t_0 (ps; fixed)	A_1	τ_1 (ps)	A_2	τ_2 (ps)
ν_{CSC} (667 cm^{-1})	-0.038 ± 0.002	11.74± 0.97	0.11 ± 0.01	2.56 ± 0.21	1.80 ± 0.22
$\nu_{S=O}$ (1043 cm^{-1})		12.79 ± 0.63		3.89 ± 0.26	

Table S5. Solvation dynamics of DMSO in the sub-bands of ν_{CSC} and $\nu_{S=O}$ modes observed in FSRS of curcumin

shared parameters (ps)				
τ_2 [a]	τ_3 [a]	τ_4 [a]		
0.72	4.44	163		
individual parameters				
vibrational mode	ω (ps)	t_0 (ps)	A_1	τ_1 (ps)
ν_{CSC} (657 cm^{-1})<br>ν_{CSC} (678 cm^{-1})	0.063 ± 0.007	0.016 ± 0.005	1.18 ± 0.20<br>-0.90 ± 0.20	0.049 ± 0.007
$\nu_{S=O}$ (1027 cm^{-1})<br>$\nu_{S=O}$ (1058 cm^{-1})	0.076 ± 0.029	0.035 ± 0.071	0.214 ± 0.759<br>-0.345 ± 1.85	0.020 ± 0.090

[a] Time constants in the population decays of the ν_{CSC} (667 cm^{-1}) and $\nu_{S=O}$ (1043 cm^{-1}) modes were used as fixed parameters.

References

1. M. J. Frisch, G. W. Trucks, H. B. Schlegel, G. E. Scuseria, M. A. Robb, J. R. Cheeseman, G. Scalmani, V. Barone, B. Mennucci, G. A. Petersson, H. Nakatsuji, M. Caricato, X. Li, H. P. Hratchian, A. F. Izmaylov, J. Bloino, G. Zheng, J. L. Sonnenberg, M. Hada, M. Ehara, K. Toyota, R. Fukuda, J. Hasegawa, M. Ishida, T. Nakajima, Y. Honda, O. Kitao, H. Nakai, T. Vreven, J. A. Montgomery, J. E. Peralta, F. Ogliaro, M. Bearpark, J. J. Heyd, E. Brothers, K. N. Kudin, V. N. Staroverov, R. Kobayashi, J. Normand, K. Raghavachari, A. Rendell, J. C. Burant, S. S. Iyengar, J. Tomasi, M. Cossi, N. Rega, J. M. Millam, M. Klene, J. E. Knox, J. B. Cross, V. Bakken, C. Adamo, J. Jaramillo, R. Gomperts, R. E. Stratmann, O. Yazyev, A. J. Austin, R. Cammi, C. Pomelli, J. W. Ochterski, R. L. Martin, K. Morokuma, V. G. Zakrzewski, G. A. Voth, P. Salvador, J. J. Dannenberg, S. Dapprich, A. D. Daniels, Farkas, J. B. Foresman, J. V. Ortiz, J. Cioslowski and D. J. Fox, Wallingford CT2009.
2. K. K. Irikura, R. D. Johnson and R. N. Kacker, *J. Phys. Chem. A*, 2005, **109**, 8430-8437.
3. J. P. Merrick, D. Moran and L. Radom, *J. Phys. Chem. A*, 2007, **111**, 11683-11700.
4. G. Batignani, G. Fumero, E. Pontecorvo, C. Ferrante, S. Mukamel and T. Scopigno, *ACS Photonics*, 2019, **6**, 492-500.
5. M. Jen, K. Jeon, S. Lee, S. Hwang, W. J. Chung and Y. Pang, *Struct. Dyn.*, 2019, **6**, 064901.
